# The Departmental Committee's Report

**Published:** 1921-06-04

**Authors:** 


### DANGEROUS DRUGS ACT NEW REGULATIONS.

### The Departmental Committee's Report.

The report of the Committee appointed by the Secretary of State for the Home Department to consider outstanding objections to the Draft Regulations under the Dangerous Drugs Act, 1920 (published in January last), has now been published, and the majority of the recommendations of that Committee have been incorporated in a revised set of Regulations, issued on May 24, 1921, by the Home Office. These will come into force on September 1 next. The report covers some seventeen pages, and it will suffice if we summarise the principal points dealt with, taking first the regulations of general application, and, secondly, those relating to the control of drugs in hospitals.

Doctor's Prescription insisted on.

The most important objections came from the British Medical Association and the Pharmaceutical Societies. The latter bodies asked that the regulation requiring a doctor's prescription for any of the drugs should be withdrawn, claiming that the register of sales kept by the requirements of the Pharmacy Acts gave ample safeguards against "doping." The Committee, however, hold the opinion that in view of the dangerous properties which the drugs in question possess they should not be issued to the public except on a doctor's prescription. It was, however, decided to recommend that certain preparations should be exempted from the operation of the Regulations —these being in common use and of such a character that there is little danger of them producing the drug habit. The British Medical Association and Pharmaceutical Society submitted, jointly, a list of such preparations, and, after consideration, the Departmental Committee has prepared a list of "exempted preparations," which appear in Schedule 2 of the revised Regulations now published. No preparation of cocaine or heroin is to be exempted.

To reduce the chance of forged prescriptions being presented to pharmacists, the Committee recommend that prescriptions containing these drugs be only given on "official forms," as in the case of National Health Insurance Prescriptions. In the event of no forms being available in emergency, ordinary prescription forms may be used if these are specially marked, and these may only be dispensed by a chemist who is acquainted with the doctor's signature or with the person for whom the prescription is written.

### Clerical Work Simplified.

In substitution for the several books required to be kept by the earlier Regulations it is agreed that the day-book or prescription-book will prove a sufficient record, with a cross index to make reference easy. Personal administration by a doctor of the drugs (such as morphia injection) is not regarded as a "supply" requiring a record. Doctor's qualifications and the words "Not to be Repeated" are not now regarded as necessary in writing prescriptions. For chronic illness a doctor may order a prescription to be repeated for not more than three times at specified intervals—an important stipulation being that each time the "repeat" is required the prescription must be made up by the same chemist.

In view of the misconception as to the wording of Regulation 13, regarding delivery by messenger, the Committee recommend that this paragraph should be altered to make it quite clear that legitimate orders can be delivered through the usual channels, *i.e.*, post, carrier, etc.

### Hospitals and Institutions.

The Committee considered the question of the application of the Regulations to hospitals and public institutions as a separate case. As we forecasted it has been decided to have a separate list of regulations for hospitals. The Home Office and representatives of some large London hospitals had provisionally agreed on a scheme to apply to the larger hospitals where the dispensary is in charge of a pharmacist, and it was reported that the scheme had been criticised on points of detail by the Pharmaceutical Society and the Public Pharmacists' Association, but, in principle, it was not questioned, and the Committee recommend its adoption. It has been necessary to deal with (*a*) hospitals where the dispensary is not in charge of a pharmacist, but of a person with the "Assistants'" certificate in dispensing of the Apothecaries' Hall; (*b*) hospitals where there is no qualified dispenser at all, but a medical officer who dispenses medicines, and (*c*) cottage hospitals with no resident medical officer.

The Committee do not regard it as necessary to include these "schemes" in the Regulations, as it may be necessary to modify them from time to time, to meet different or fresh circumstnaces, and they proposed that the Regulations provide for the exemption by the Secretary of State from the operation of the Regulations of any hospital or public institution, subject to the observance of such conditions as he shall by order prescribe. It was pointed out that at Poor-law institutions persons with the "Assistants'" certificate in dispensing (who are not pharmacists) are eligible to act as "Dispensers," and, unless provision was made for authorising persons now acting, their position would be prejudiced. As these appointments have to be approved by the Ministry of Health and the institutions are inspected by the Ministry the Committee recommended, justly, we think, that these dispensers should be made "Authorised" persons. In all other institutions without such safeguards it is recommended that reasonable experience, say three years as dispenser-in-charge, should be required in addition.

### The Scheme for Control of the Drugs in Hospitals.

This is published as an appendix to the report in three sections. (1) Where the dispensary is in charge of a qualified pharmacist the supplies are to be received and kept by the pharmacist, and a record made in the drug-ledger. The supplies for ward "Stock" use are to be kept in a locked cupboard by the sister-in-charge, and used as ordered by the doctor. These supplies may only be obtained by *written* requisition of the sister-in-charge in a counterfoil book—the counterfoil to be initialled by the dispenser and a record kept in the dispensary of all such supplies to the wards.

Prescriptions for individual patients are to be written on the bed-cards by the doctor attending, and *initialled* by him. Records are to be kept in the dispensary of the fact that prescriptions have been dispensed by stamping the card, and these will form the reference.

An "Index Book" is to be kept at the dispensary showing the name or reference number of the patient, doctor's name, date. This replaces the requirement in former regulations for a *separate* prescription (to be retained at the dispensary), the doctor's full name and qualifications and the words "Not to be Repeated," and should be comparatively easy to observe.

A similar scheme is proposed for (*a*) Poor-law Institutions, where the dispenser has a qualification recognised by the Ministry of Health and the appointment has been approved by the Ministry (*b*) For other *bonâ-fide* public institutions approved by the Secretary of State as do not employ a pharmacist and to whom exemption may properly be granted who employ as sole or head dispenser a person who has a qualification recognised by the Ministry of Health for Poor-law purposes, and, in addition, has had three years' experience as sole or head dispenser at such an institution.

### Points to be Noted.

The foregoing scheme should be fairly adaptable to the different classes of institutions, and certainly is much more suitable and workable than the original Regulations issued. There appears to be a divergence of opinion amongst some hospital officials as to the soundness of the clause allowing the ward sister to obtain, on her own requisition, supplies of morphia injection, etc. At most Poor-law, fever, and mental hospitals all these requisitions are signed by the resident medical officer as an additional safeguard. Indeed, it is doubtful now whether an order for "Stock" supply (as distinct from a prescription) can be supplied on the signature of a medical officer or if the ward sister is the only person who can sign the order in her "counterfoil" book.

It is to be noted that, in future, all hospitals and institutions requiring a dispenser to have full charge of the dispensary and supply morphia, etc., *must* appoint either a pharmacist or a person with the other qualifications mentioned (assistants' certificate) who has, in addition, been in sole charge of a dispensary at a *bonâ-fide* public institution for three years.

